# Cortical contusion injury disrupts olfactory bulb neurogenesis in adult mice

**DOI:** 10.1186/1471-2202-14-142

**Published:** 2013-11-13

**Authors:** Kryslaine L Radomski, Qiong Zhou, Kevin J Yi, Martin L Doughty

**Affiliations:** 1 Center for Neuroscience and Regenerative Medicine (CNRM), Department of Anatomy, Physiology and Genetics, Uniformed Services University of the Health Sciences, 4301 Jones Bridge Road, Bethesda, MD 20814, USA

**Keywords:** Traumatic brain injury (TBI), Controlled cortical impact (CCI), Subventricular zone (SVZ), Neural stem cell (NSC), Olfactory bulb

## Abstract

**Background:**

Experimental brain trauma activates quiescent neural stem cells (NSCs) to increase neuronal progenitor cell proliferation in the adult rodent brain. Previous studies have shown focal brain contusion in the form of a unilateral controlled cortical impact (CCI) stimulates NSCs to bilaterally increase neurogenesis in the adult hippocampus.

**Results:**

In this study we clarified the bi-lateral effects of a unilateral CCI on proliferation in the subventricular zone (SVZ) NSC niche and on neurogenesis in the olfactory bulb of adult mice. By varying the depth of impact from 1 mm to 2 mm depth, we show CCI to the left somatosensory cortex resulted in graded changes in mouse behavior and cellular pathology in the forebrain. As expected, contusion to the sensorimotor cortex resulted in motor coordination deficits in adult mice. During the first 3 days after injury, CCI increased proliferation in the impacted cortex, deeper striatum and SVZ of the forebrain ipsilateral to the CCI. In each of these regions proliferation was increased with increasing injury severity. At 30 days post-procedure, CCI resulted in a significant reduction in neurogenesis in the olfactory bulb ipsilateral to the CCI. Olfactory avoidance testing indicated disruptions in olfactory bulb neurogenesis were associated with impaired olfactory discrimination in mice post-injury.

**Conclusion:**

The data demonstrate a focal cortical contusion injury to the left somatosensory cortex disrupts SVZ-olfactory bulb neurogenesis and impairs olfactory discrimination and motor coordination in adult mice.

## Background

Traumatic brain injury (TBI) initiates a complex cascade of cellular events in the adult brain resulting in cognitive and behavioral deficits [1]. Significantly, TBI is generally followed by a spontaneous recovery process that diminishes these functional deficits over time [2]. The partial or complete recovery of function underlies the plasticity of the brain and functional imaging studies have confirmed the ability of neural circuits to reorganize post-injury to recapitulate function [3]. Although it remains debatable whether functional recovery involves the production of new neurons in the adult brain [4], it is established that many forms of experimental brain trauma activate quiescent neural stem cells (NSCs) to increase neuronal progenitor cell proliferation in the adult rodent brain [5-8].

Controlled cortical impact (CCI) is a graded, focal contusion model of experimental TBI commonly used in rodents [9]. CCI in adult mice activates NSCs to increase proliferation in both the hippocampus and SVZ neurogenic niches [8,10]. Cell lineage studies using transgenic mice indicate CCI evokes a sustained increase in neurogenesis in the adult hippocampus and exerts differential effects in hippocampal NSCs depending on their state of lineage progression. CCI depletes intermediate progenitor cell (IPC) populations and simultaneously activates radial glial-like (RGL) nestin-expressing type-1 multi-potent NSCs to divide and repopulate the lost IPCs [10]. Targeted ablation of dividing nestin-expressing RGL cells inhibits the repopulation of IPCs following CCI and exacerbates the cognitive effects of CCI in mice, revealing a functional role for injury-induced neurogenesis in the hippocampus [11].

The effects of focal head trauma in the rodent SVZ are less defined [7]. There is general agreement trauma to the cerebral cortex results in transient increases in cell proliferation in the adult SVZ [5]. Whether this occurs in the SVZ of both cerebral hemispheres remains uncertain [8,12,13] and it is unclear what the effects of TBI-induced changes in the SVZ have on neurogenesis in the adult olfactory bulb [14-16]. Neuroblasts have been reported to migrate ectopically from the SVZ to the damaged cortex in adult TBI mice at the expense of the olfactory bulb [8,17] but this phenomenon has only been observed transiently in the acute (3–4 days) post-injury period and was not reported to result in sustained neurogenesis deficits in the adult olfactory bulb [17].

In this study we sought to clarify the effects of focal cortical injury on neurogenesis in the adult olfactory bulb. We examined the bi-lateral effects of graded, unilateral CCI on proliferation in the SVZ and neurogenesis in the granular cell layer of the olfactory bulb using unbiased stereological counting techniques. Our data reveals CCI in adult mice significantly increases proliferation in the SVZ of the cerebral hemisphere ipsilateral to the CCI in the acute (3-day) post-injury period. Proliferation increased with increased CCI severity in both the ipsilateral and contralateral SVZ but was statistically significant for only the severest 2 mm depth CCI ipsilateral. In contrast to increases in SVZ proliferation, 2 mm and 1 mm depth CCI resulted in a significant decrease in neurogenesis in the granule cell layer of the olfactory bulb ipsilateral but not contralateral to the CCI one month post-injury. Olfactory avoidance testing reveals CCI results in acute deficits in olfactory behavior in mice. This behavioral deficit diminishes with time post-injury at a rate relative to CCI severity with deficits persisting in mice subjected to the severest CCI one month post-injury. These data demonstrate focal cortical contusion disrupts olfactory behavior and impairs SVZ neurogenesis to the olfactory bulb in the cerebral hemisphere ipsilateral but not contralateral to a unilateral CCI in adult mice.

## Methods

### Animals

Experiments were performed on 8–10 week-old adult C57BL/6 J male mice obtained from The Jackson Laboratory, Bar Harbor, ME. All procedures were approved by the Uniformed Services University of the Health Sciences Institution for Animal Care and Use Committee (IACUC). Mice were housed in the Uniformed Services University’s Center for Laboratory Animal Medicine on a standard 12 hour light–dark cycle, with feed and water available *ad libitum*.

### Controlled cortical impact (CCI)

Mice were placed under anesthesia in an induction chamber containing a mixture of O_2_ and isoflurane (2-4%) delivered by a vaporizer. Anesthesia was maintained during procedures by a mixture of O_2_ and 0.25-2% isoflurane using a nosecone (Dan Kopf Instruments). Mice were checked for adequate anesthesia by lack of response to toe-pinch. Core mouse body temperature was monitored by a thermometer probe and maintained at 36–38°C on a warming pad. The head was placed in a stereotactic device and held in a horizontal plane by ear and incisor bars (Stoelting). The fur was removed from the intended impact area of the cranium using an electric razor. The shaved scalp was sterilized with Betadine and 70% ethanol and the skull exposed using a small ≈ 10 mm surgical incision over the scalp. A 5.0 mm burr hole was drilled into the skull with a hand-held trephine to expose the dura mater. The impact tip (3 mm in diameter) was gently lowered to the surface dura to register 0 mm depth on the CCI electromagnetic controller (Impact One, Leica). A single contusion of 1 mm or 2 mm depth was then made to the somatosensory cortex (coordinates 0 mm Bregma, 2 mm lateral left) at an impactor velocity of 1.5 m/s with a dwell time of 100 ms and an impact angle of 15º. Sham treated animals were given a 5.0 mm burr hole craniotomy as described but not impacted. The incision was closed with sutures following CCI or sham procedure. Naïve animals were anesthetized as described for those undergoing surgery but were not placed in the stereotaxic frame and did not undergo scalp incision or craniotomy. After the procedure, the animals were returned to a cage placed on a heating pad and monitored continually until they were alert and ambulating. Recovery time was typically less than 10 minutes. Mice were housed singularly after the procedure and did not receive analgesics that could confound post-procedure behavioral measures.

### BromodeoxyUridine (BrdU) injections

Mice were given intraperitoneal injections of 100 mg/Kg body weight BromodeoxyUridine (BrdU) in saline. A single BrdU injection was administered at 10 minutes and at 24, 48 and 72 hours post-CCI/sham procedure. Naïve mice were injected at equivalent times. Mice analyzed for acute proliferative responses in the SVZ and forebrain parenchyma were perfusion-fixed 2 hours after the last BrdU injection (74 hours post-CCI/sham procedure). Mice analyzed for chronic cell fate in the olfactory bulb and forebrain were sacrificed by perfusion-fixation at 30 days post-CCI/sham procedure.

### Behavioral testing

Baseline measurements were conducted for all mice used in behavioral studies 24 hours before the CCI/sham procedure followed by testing on days 1, 10 (except for rotarod test) and 29 post-CCI/sham. Naïve animals were tested on equivalent times. All behavioral testing was performed by a single investigator blinded to injury severity. *Open field test*. Gross motor ability and exploratory behavior was assessed using the open-field test. The mouse was placed in the center of a square 40 cm × 40 cm open field apparatus with black opaque walls (Stoelting) and permitted to explore the surroundings for 10 minutes. An overhead camera linked to a computer with ANY-maze behavioral tracking software (Stoelting) tracked and recorded the mouse’s movements and reported the total distance traveled during the test session for each mouse. *Accelerating Rotarod test.* The rotarod test was used to evaluate sensorimotor deficits. Mice were acclimated to the test by being placed on a rotating rod (Ugo Basile) for 240 seconds at a constant speed of 4 rpm prior to each testing session. Testing consisted of recording the animal’s mean latency to fall from the rotating rod set to accelerate from 4 to 40 rpm over 300 seconds. Each animal underwent 4 trials with a 3-min rest period in between each trial. *Olfactory avoidance test*. This behavioral paradigm has been described as a sensitive test to detect deficits in the ability of mice to sense an aversive scent, such as acetic acid [18]. Briefly, the ANY-maze software was used to record the mouse’s movements within the open field apparatus in relation to filter paper squares (5 cm × 5 cm) soaked with either 5% acetic acid or distilled water (used as an odorless control). After habituating the mouse to the testing arena for 10 min, a filter paper scented with water was introduced to a corner of the open field square chamber and the duration each mouse spent investigating the filter paper was recorded during a 3 minute test period. The water-impregnated filter paper was then removed and after a 1 min interval it was replaced by a filter paper scented with acetic acid. The time each mouse spent investigating the odorant-scented filter paper was similarly recorded for 3 min. A positive investigation was defined by any time the mouse’s nares were in immediate proximity (<1 mm) of the filter paper. The time each mouse spent investigating the water-scented paper was subtracted from the value obtained with the acetic-acid odor to give the relative avoidance time.

### Immunohistochemistry (IHC)

Mice were placed under anesthesia (as described for CCI surgery) and transcardially perfused with ice-cold phosphate buffered saline (PBS) followed by 4% paraformaldehyde in PBS. The brain was dissected out and immersion fixed in fresh fixative overnight at 4°C. The brain was washed in PBS and embedded in agarose for sectioning on a vibrating VT-1000 vibratome (Leica). Brains were sectioned at 40 μm thickness in either the coronal or sagittal plane. Serial sections were stored in PBS in 96-well plates at 4°C until use. For anti-BrdU IHC, sections were incubated for one hour at room temperature (RT) in 2 N HCl and the acid neutralized by 0.1 M Borate buffer. For all IHCs, sections were blocked in 10% normal goat serum (NGS) in PBS for 1 hour at RT. Sections were incubated free-floating in primary antibodies overnight in PBS containing 1% NGS, 0.2% Triton X-100. Details of the primary antibodies used are as follows: (1) 1: 500 dilution of rat monoclonal anti-BrdU (Accurate Chemical & Scientific Corp.); (2) 1: 1,000 dilution of rabbit polyclonal anti-Glial Fibrillary Acidic Protein (anti-GFAP, Millipore); (3) 1: 1,000 dilution of rabbit polyclonal anti-Iba1 (Wako Pure Chemical Industries Ltd.); (4) 1: 500 dilution of mouse monoclonal anti-NeuN (clone A60, Millipore). Primary antibody combinations for dual IHC were incubated and processed simultaneously. Immunolabeled sections were washed in PBS and incubated with Alexa Fluor ® 488, 555 or 647 dye-labeled secondary antibodies (Life Technologies) at 1:1,000 dilutions in PBS for 60 minutes at RT, counterstained with DAPI and mounted under Mowiol. Immunofluorescent images were captured on a Zeiss A1 or M1 (stereology only) Imager microscopes with Axiocam digital cameras, or on a Zeiss Pascal laser scanning confocal microscope.

### Stereological cell counting procedures

Unbiased stereological methods were conducted on blind-coded slides by a single investigator blinded to the injury status of the animals. All stereological cell counts were performed using MicroBrightField Bioscience Stereo Investigator software linked to a Zeiss M1 imager microscope with a motorized x, y and z-plane stage. The total number of anti-BrdU immunolabeled cells (BrdU+) cells in the SVZ and dual anti-BrdU/anti-NeuN (BrdU+/NeuN+) immunolabeled cells in the granule cell layer of the olfactory bulb were estimated using the unbiased Optical Fractionator probe of the StereoInvestigator program. Stereological cell counts were obtained from both cerebral hemispheres ipsilateral and contralateral to the CCI/sham (a single hemisphere was counted for naïve controls). To determine the number of BrdU immunolabeled cells in the lateral wall of the SVZ, 6 sections (240 μm apart), starting at approximately 1.46 mm rostral to Bregma, were selected from each animal and processed for anti-BrdU IHC as described. Systematic random sampling areas (137 μm × 104 μm) in each section through the anterior portion of the SVZ were randomly chosen by the StereoInvestigator program with a counting frame area of 50 μm × 50 μm. Section thickness was determined by marking when DAPI-counterstained cells first appeared in focus. Z series of 30 μm depth with 1 μm intervals between images were collected. BrdU+/DAPI + nuclei that first appeared in focus within the 20 μm Z-stack counting probe were counted. In the olfactory bulb, the number of BrdU/NeuN double-positive cells in the granular cell layer was counted on 5 sagittal sections (160 μm apart). Positive profiles were counted within a 60 μm × 60 μm counting frame spaced in a 300 μm × 300 μm sampling grid with a 30 μm Z-stack counting probe. Dual anti-BrdU/anti-NeuN immunolabeled cells were defined by co-localization of the two immunofluorescent signals in the same z-plane. All together, the Gundersen (m = 1) coefficient of error ranged between 0.06 and 0.12.

### Non-stereological cell counting procedures

Counts of dual anti-BrdU/anti-GFAP, dual anti-BrdU/anti-Iba1, or dual anti-BrdU/anti-NeuN cells were made from digitally captured images from peri-contusional areas of the neocortex and the striatum. Counts were made from the cerebral hemisphere ipsilateral to the CCI or sham only as minimal anti-BrdU labeling was observed in non-neurogenic regions in the contralateral cerebral hemisphere (as well as from a single cerebral hemisphere in naïve mice). In each case, z-stacks or wide-field z-plane series fluorescent images were collected at 20× magnification from peri-contusional areas and the number of dual immunofluorescent cells present within the image area recorded. Cells were counted if the whole nucleus was inside the image area.

### Measurement of the lesion cavity volume

For analysis of lesion volume, nine coronal sections at 440 μm intervals spanning the extent of the injury were processed for DAPI immunohistochemistry and imaged using a Hamamatsu Nanozoomer 2.0-RS digital slide scanner. Measurements of cortical lesion area in each section were performed using the ImageJ software. The total volume of the cortical lesion was calculated by multiplying the area obtained from each section by the distance between each section.

### Data and statistics

All histograms are plotted as mean plus standard error of the mean (SEM) error bar above. Multiple comparisons were analyzed using repeated measures one-way ANOVA with post hoc Tukey’s test unless otherwise stated. P-values < 0.05 were considered significant. Unless otherwise stated, p-values stated are from Tukey’s test comparisons to naïve controls.

## Results

### CCI results in graded acute and chronic behavioral changes in adult mice

**Figure 1:**
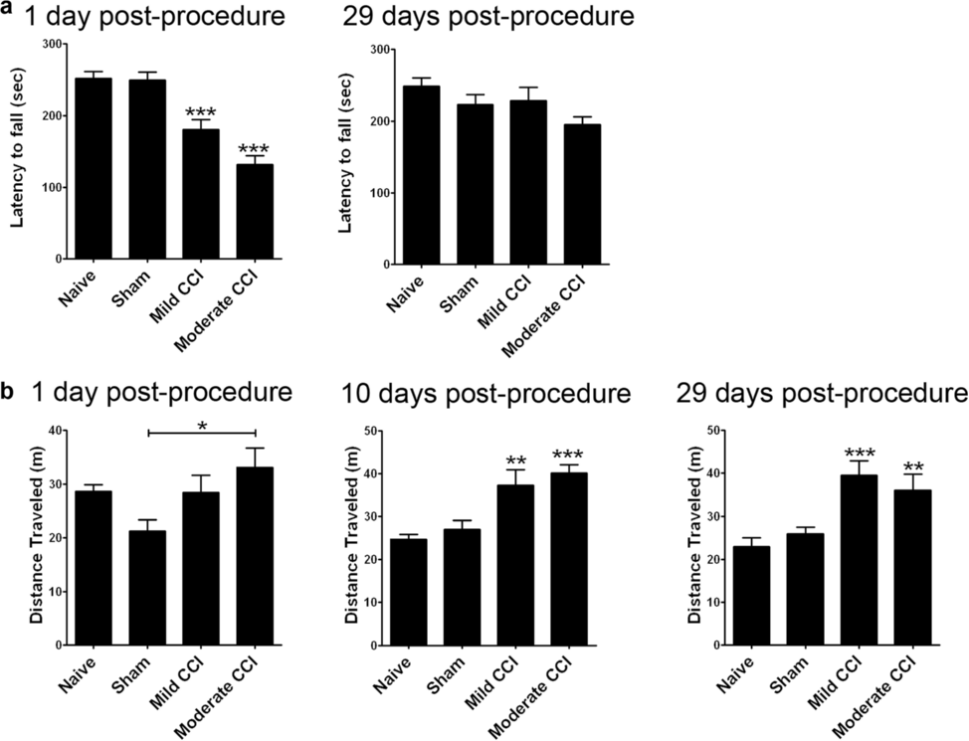
**Unilateral CCI to the somatosensory cortex impairs sensorimotor coordination and triggers locomotor hyperactivity in adult mice. (a)** CCI significantly impairs sensorimotor coordination at 1 day post-procedure as measured by the latency to fall from an accelerating rotarod (*** p < 0.001 moderate CCI versus naïve and mild CCI versus naïve). Accelerating rotarod testing at 29 days post-procedure reveals a recovery in sensorimotor coordination in moderate and mild CCI mice. The mean scores show a gradation of sensorimotor impairment with injury severity with moderate CCI mice performing worse than mild CCI. **(b)** CCI triggers locomotor hyperactivity in adult mice. There is no significant difference between CCI mice and naive mice at 1 day post-procedure but moderate CCI mice did travel significantly greater distances when compared to sham at this acute time point (* p < 0.05). Mild and moderate CCI mice travel significantly greater distances in open-field testing at 10 days post-procedure (*** p < 0.001 moderate CCI versus naïve, * p < 0.05 mild CCI versus naïve) and 29 days post-procedure (* p < 0.05 moderate CCI versus naïve, *** p < 0.001 mild CCI versus naïve). The performance of shams was not significantly different from naïve on either test at any post-procedure time point indicating these behavioral changes were due to the effects of cortical contusion. Data shown as mean ± SEM with n = 12/group, except open-field testing at 10 days post-procedure n = 10/group.

The goal of this study was to measure the effects of increasing severity of a cortical contusion injury on neurogenesis from the subventricular zone (SVZ) of adult mice. For contusion, we used 1 mm and 2 mm depth controlled cortical impact (CCI) to the left somatosensory cortex of 8–10 week-old C57BL/6 J mice. These two CCI paradigms are referred to as mild (1 mm depth) and moderate (2 mm depth) CCI throughout this manuscript. In order to determine if mild and moderate CCI resulted in graded post-injury responses in mice, we compared motor performance in mice post-procedure using the accelerating rotarod. We reasoned CCI to the somatosensory cortex would lead to specific deficits in the coordination of motor skills examined by this test. As expected, at 24 hours post-procedure rotarod performance was significantly compromised in mild CCI (p < 0.001) and moderate CCI (p < 0.0001) but not sham mice (Figure 1a). This acute reduction in rotarod performance was proportional to CCI severity. Although both CCI groups exhibited improved rotarod performance at 29 compared to 1 day post-CCI/sham, such that their performance was no longer statistically different from naive, moderate CCI mice continued to exhibit a greater mean deficit in this task when compared to mild CCI mice (Figure 1a).

To confirm these results were specific to a lack of coordination and not a paucity of movement in mice, we used the open field test to measure unconditioned motor activity. This test revealed CCI did not result in acute differences in locomotor activity when compared to naïve mice at 1 day post-procedure (Figure 1b). However, when compared to sham controls at this acute post-injury stage, moderate CCI mice showed increased locomotion (total distance traveled) (p < 0.05, Figure 1b). This trend of increased activity persisted and total locomotor activity significantly increased with mild and moderate CCI at 10 days (p < 0.01 and p < 0.001 respectively, Figure 1b) and 29 days post-procedure (p < 0.001 and p < 0.05 respectively, Figure 1b). These results demonstrate CCI to the somatosensory cortex results in acute deficits in motor coordination proportional to contusion depth and sustained locomotor hyperactivity in mice. Combined these tests confirm our CCI paradigms result in graded acute and chronic behavioral changes in adult mice.

### CCI increases proliferation in the cerebral hemisphere ipsilateral to the CCI/sham

**Figure 2:**
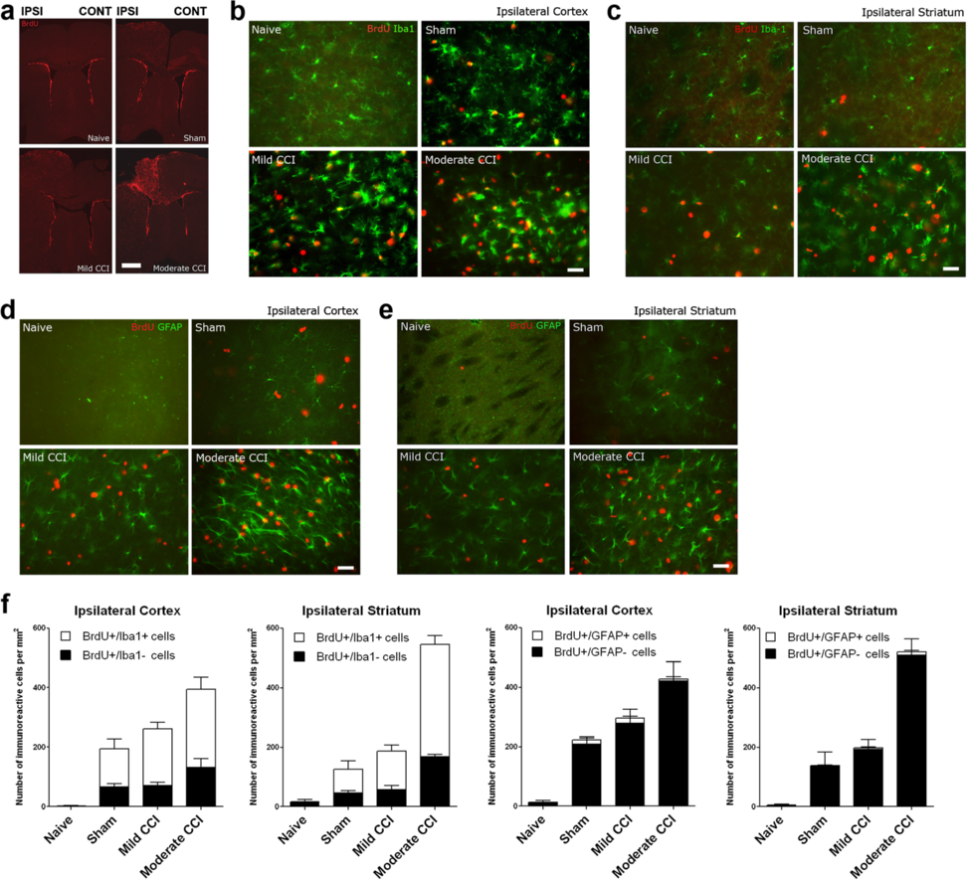
**Unilateral trauma to the somatosensory cortex increases cell proliferation in the cerebral hemisphere ipsilateral to the CCI/sham. (a)** Short-term BrdU incorporation analysis reveals widespread activation of cell proliferation in the cerebral hemisphere ipsilateral to the CCI or sham craniotomy. Mice were processed for anti-BrdU IHC at 74 hours post-procedure following BrdU injections at 0, 24, 48 and 72 hours post-procedure. In each image panel the cerebral hemisphere ipsilateral (IPSI) to the CCI/sham is on the left, the contralateral (CONT) hemisphere is on the right. Scale bar = 1 mm. (b and c) Co-immunolabeling of anti-Iba1 antisera (green), a marker for microglia and macrophages, with anti-BrdU + (red) reveals numerous co-immunoreactive cells in the **(b)** cortex and **(c)** striatum of the cerebral hemisphere ipsilateral to the CCI or sham craniotomy. Scale bars = 20 μm. (d and e) Co-immunolabeling with anti-GFAP antisera (green), a marker for reactive astrocytes, reveals astrogliosis in the **(d)** cortex and **(e)** striatum of the cerebral hemisphere ipsilateral to the CCI or sham craniotomy but minimal astrocyte contributions to the BrdU + cell population (red). Scale bars = 20 μm. **(f)** Quantification of immunolabeled cell numbers in the ipsilateral cortex and striatum reveals a graded increase in BrdU + cell numbers with increasing injury severity. Co-immunoreactivity to anti-Iba1 antisera confirms the majority (>50%) of these BrdU + cells are proliferating microglia or macrophages in CCI and sham mice, whereas astrocytes account for only 2-7% of the BrdU + cell population (n = 4-12/group).

Having established behavioral responses to our CCI paradigm, we next examined forebrain histopathology in CCI, sham and naïve mice. Mice were examined at 74 hours post-CCI/sham procedure following daily injections of BrdU immediately after surgery and at 24, 48 and 72 hours post-procedure (Figure 2). Anti-BrdU IHC revealed extensive proliferation in the cerebral hemisphere ipsilateral to the CCI during the acute recovery period. Anti-BrdU immunoreactive (BrdU+) cells were distributed throughout the medial-lateral, and dorso-ventral axis of the cerebral hemisphere with highest density in the peri-contusional areas of the primary and secondary somatosensory and motor cortices (Figure 2a). The density of BrdU + cells in the cerebral hemisphere contralateral to the CCI procedure was considerably lower, although there was some clustering of BrdU + cells in adjacent medial areas of the primary and secondary motor cortex and, to a lesser degree, the septum (Figure 2a). Co-immunolabeling of antibodies to the microglia/macrophage marker Iba1 revealed numerous anti-Iba1 co-immunoreactive BrdU + cells in the cortex (Figure 2b) and striatum (Figure 2c) of the cerebral hemisphere ipsilateral to CCI. In comparison, immunolabeling with antibodies to glial fibrillary acidic protein (GFAP), a marker of reactive astrocytes, revealed higher numbers of GFAP + cells in the ipsilateral cortex (Figure 2d) and striatum (Figure 2e) following CCI, although minimal BrdU + cells co-immunolabeled with the anti-GFAP antisera.

Quantification of the IHC data confirms the majority of post-injury proliferating cells in the peri-contusional areas of the ipsilateral cortex and striatum were Iba1-expressing microglia/macrophages, with minimal contribution to the proliferating cell pool from GFAP-expressing astrocytes (Figure 2f). Of note, quantification revealed a graded response to injury in the ipsilateral cerebral hemisphere with the density of proliferating BrdU + cells increasing with increasing injury severity from sham to mild to moderate CCI (Figure 2f). Hence, both behavioral and histological assessments of our CCI/sham mice reveal graded functional and histopathological changes in response to increasing injury severity.

### Increased SVZ proliferation in the cerebral hemisphere ipsilateral to the CCI

**Figure 3:**
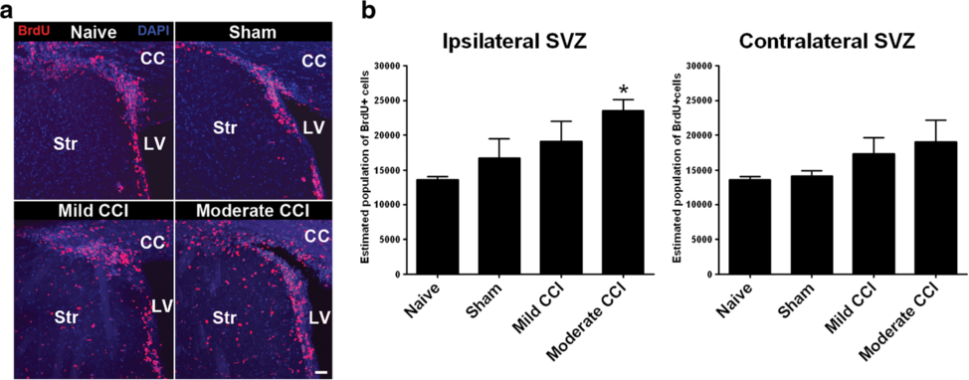
**Acute increases in cell proliferation in the subventricular zone (SVZ) of the cerebral hemisphere ipsilateral to a CCI. (a)** Short-term BrdU incorporation analysis identifies proliferating cells, including mitotically active neural stem cells, in the SVZ lining the lateral wall of the forebrain lateral ventricle. Mice were processed for anti-BrdU IHC at 74 hours post-procedure following BrdU at 0, 24, 48 and 72 hours post-procedure. The images were taken from the SVZ of coronal sections of the cerebral hemisphere ipsilateral to the CCI/sham. Key: CC, corpus callosum; LV, lateral ventricle; Str, striatum. Scale bar = 20 μm. **(b)** Stereological cell counts reveal a significant increase in the number of BrdU-incorporated cells in the SVZ of the cerebral hemisphere ipsilateral but not contralateral to a moderate CCI (* p < 0.05 moderate CCI versus naïve [n = 4/group], one-way ANOVA with Tukey’s multiple comparison post test). Although the data reveals a trend of increasing BrdU + cell numbers in both SVZs with increasing injury severity, no other increases were significant when compared to naïve.

We next quantified acute (74 hour post-procedure) effects of mild and moderate CCI on proliferation in the forebrain SVZ using the same 4-day BrdU injection regimen (Figure 3). Anti-BrdU IHC revealed clusters of BrdU + cells in the lining the lateral wall of the lateral ventricles (Figure 3a), a distribution of BrdU + cells that is consistent with the labeling of mitotically active neural stem cells in the SVZ stem cell niche. We used stereological cell counting procedures to quantify the number of BrdU + cells in the lateral walls of the lateral ventricles in the cerebral hemisphere ipsilateral and contralateral to the CCI/sham. This analysis revealed a graded effect of injury on SVZ proliferation (Figure 3b), consistent with the effects of CCI/sham on proliferation in the cortex and striatum. Although proliferation increased with increasing injury severity in the SVZ of both cerebral hemispheres, the increase was only significant (p < 0.05) for the moderate 2 mm CCI procedure in the ipsilateral forebrain. Moderate CCI resulted in an acute 1.73-fold increase in the number of anti-BrdU immunolabeled cells in the ipsilateral SVZ compared to naïve mice.

### Reduced olfactory bulb neurogenesis in the cerebral hemisphere ipsilateral to the CCI

**Figure 4:**
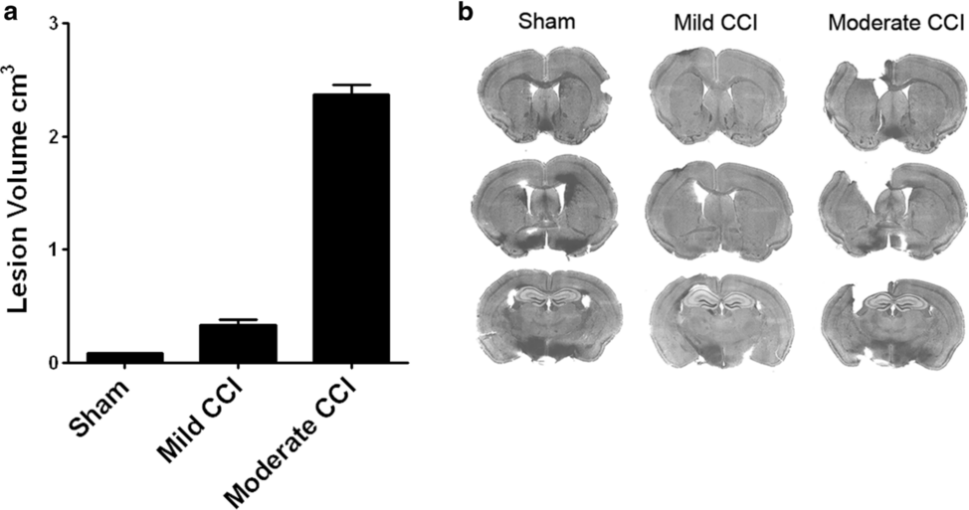
**CCI results in cortical tissue loss at 30 days post-procedure that increases with increasing injury severity. (a)** Lesion volume was measured from 9 serially-spaced coronal forebrain sections taken from sham, mild and moderate CCI mice. Data shown as mean ± SEM with n = 3/group. **(b)** DAPI counterstained coronal forebrain sections from sham, mild and moderate CCI mice sacrificed at 30 days post-procedure.

In order to determine if acute increases in SVZ proliferation resulted in long-term changes in neurogenesis, we examined the fate of BrdU-pulse labeled cells in the olfactory bulb at 30 days post-procedure. Mice sacrificed at this time point exhibited gross signs of CCI-induced losses of cortical tissue with a visible cavity present at the surface of the left somatosensory cortex in all moderate and mild CCI mice. To determine the size of the lesion we measured the volume of this cavity in 9 serially-spaced coronal forebrain sections taken from sham, mild and moderate CCI mice (Figure 4). These measurements revealed a lesion size of 2.37 ± 0.08 cm^3^ in moderate CCI mice, a volume 7-fold greater than the lesion in mild CCI mice (0.34 ± 0.04 cm^3^) and 30-fold greater than the shallow lesion present in sham mice (0.08 ± 0.002 cm^3^).

Neuroblasts generated in the SVZ migrate to the olfactory bulb along the rostral migratory stream (RMS) where they differentiate into interneuron subtypes [19]. In adult mice, the vast majority of surviving SVZ neuroblasts differentiate into granule cell interneurons in the deeper granular cell layer [20,21]. Accordingly, we used stereological cell counting methods to quantify the numbers of BrdU-pulse labeled neurons in the granular cell layer identified by dual anti-BrdU/anti-NeuN IHC. Mice were injected with BrdU at 0, 24, 48 and 72 hours post-procedure as before and processed for dual IHC 30 days post-procedure.

**Figure 5:**
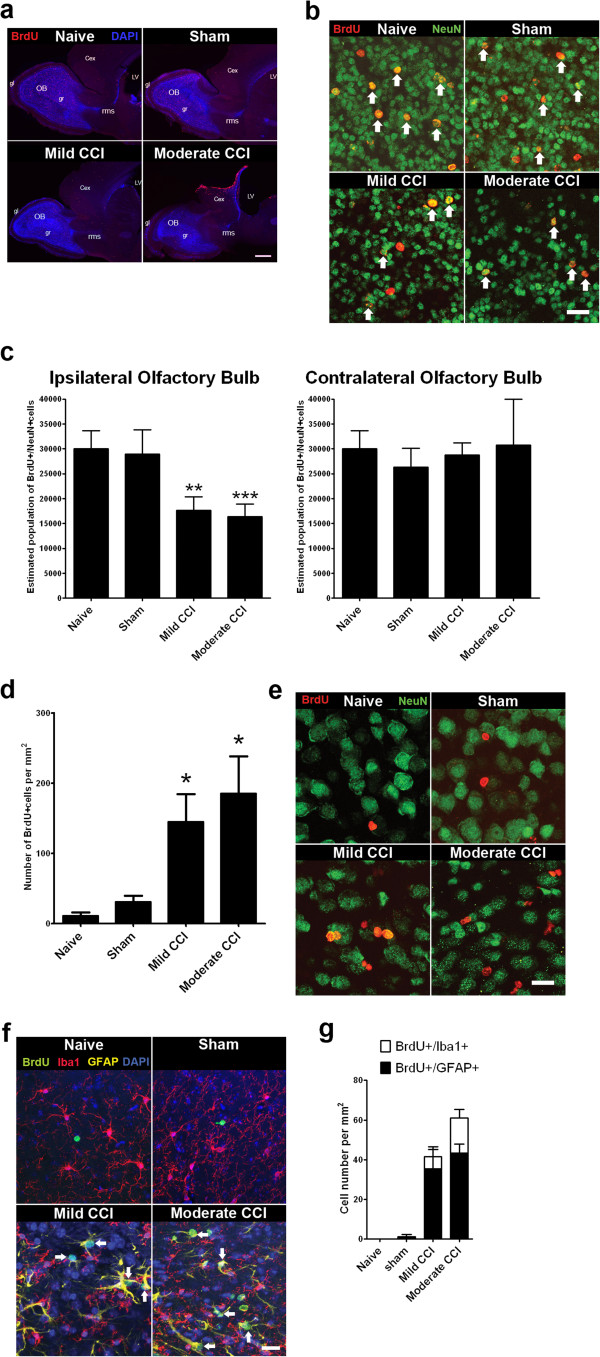
**Adult neurogenesis is decreased in the olfactory bulb of the cerebral hemisphere ipsilateral to CCI. (a)** Long-term BrdU incorporation analysis identifies olfactory bulb interneurons generated from adult neural stem cells in the SVZ of adult mice. Sagittal sections processed for anti-BrdU IHC at 30 days post-procedure following BrdU at 0, 24, 48 and 72 hours post-procedure. BrdU + cells are distributed in the granular cell layer (gr) and glomerular layer (gl) of the olfactory bulb (OB) and rostral migratory stream (RMS) passing between the cortex (Cex) and striatum (Str). Scale bar = 200 μm. **(b)** Higher magnification images from sagittal sections of the olfactory bulb ipsilateral to the CCI or sham craniotomy. Dual anti-BrdU/anti-NeuN immunolabeled neurons (arrows) in the olfactory bulb granular cell layer consistent with BrdU-incorporation in olfactory bulb-fated SVZ neural stem cells. Scale bar = 20 μm. **(c)** Stereological cell counts reveal a significant decrease in the numbers of BrdU-incorporated, NeuN immunopositive neurons in the granular cell layer in the olfactory bulb ipsilateral to moderate and mild CCI at 30 days post-injury (*** p < 0.001 moderate CCI versus naïve, ** p < 0.01 mild CCI versus naïve [n = 4/group], one-way ANOVA with Tukey’s multiple comparison post test). **(d)** Cell counts reveal an increase in BrdU-incorporated cells in the neocortex ipsilateral to moderate and mild CCI at 30 days post procedure (* p < 0.05 versus naïve [n = 5/group], one-way ANOVA with Tukey’s multiple comparison post test). **(e)** BrdU + cells in the injured cortex do not express NeuN. Scale bar = 20 μm. **(f)** Triple anti-BrdU/anti-GFAP/anti-Iba1 IHC reveals dual BrdU + /GFAP + cells (arrows). Scale bar = 20 μm **(g)** Quantification confirms many BrdU-incorporated cells have acquired a GFAP + astrocytic fate in the injured cortex 30 days post-injury.

BrdU immunolabeling at 30 days post-procedure revealed a majority of cells distributed in sites consistent with BrdU incorporation in mitotically active neural stem cell lineages. BrdU + cells were distributed in the RMS and olfactory bulb in naïve, sham and CCI mice (Figure 5a). The majority of BrdU + cells were present in the olfactory bulb granular cell layer, with BrdU + cells also found in lower densities in the olfactory bulb glomerular layer and RMS (Figure 5a), as well as in the hippocampus dentate gyrus (not shown). Confocal microscopy confirmed BrdU + cells in the olfactory bulb granular cell layer were co-immunolabeled with anti-NeuN antisera (NeuN+) identifying them as neurons (Figure 5b). Stereology revealed a significant decrease in the numbers of dual BrdU+/NeuN + cells in the granular cell layer of the ipsilateral olfactory bulb of mild (p < 0.01) and moderate CCI mice (p < 0.001) compared to naïve controls (Figure 5c). This reduction was also significant when compared to sham mice (p < 0.01 mild and moderate CCI, 1-way ANOVA) confirming a direct, deleterious effect of cortical contusion on adult olfactory bulb neurogenesis. In contrast, olfactory bulb granular cell layer neurogenesis was unaffected in the contralateral cerebral hemisphere (Figure 5c). Comparisons of BrdU+/NeuN + numbers in the ipsi- and contralateral olfactory bulbs revealed a 1.63-fold reduction in new neurons with mild CCI and a 1.87-fold reduction with moderate CCI. Consequently, stereology demonstrates focal cortical contusion stimulates SVZ proliferation but substantially reduces olfactory bulb neurogenesis in the ipsilateral cerebral hemisphere.

Cell counts in the ipsilateral cortex reveal reductions in olfactory bulb neurogenesis were associated with a corresponding increase in the presence of BrdU + cells in peri-contusional cortical areas in CCI mice at 30 days post-procedure (p < 0.05, Figure 5d). Co-immunolabeling with anti-NeuN antisera revealed negligible numbers of dual NeuN+/BrdU + cells (< 1% of BrdU+) in the cortex of all animal groups (Figure 5e). In contrast, BrdU + cells in the ipsilateral cortex of CCI mice coimmunolabel with anti-GFAP or anti-Iba1 antisera (Figure 5f), confirming a non-neuronal fate of many BrdU + cells at 30 days post-injury. Cell counts reveal more BrdU + cells are GFAP + (35.3 ± 9.9 in mild CCI and 43.3 ± 4.6 in moderate CCI mice) than Iba1+ (6.2 ± 5.1 cells in mild CCI and 17.7 ± 4.3 in moderate CCI mice) in CCI mice at this stage (Figure 5g). These results indicate a significant fraction of the surviving cells labeled with BrdU during the acute post-injury period have acquired an astrocytic fate by 30 days post-injury.

### CCI results in deficits in olfactory avoidance behavior that recover at a rate proportional to CCI severity

**Figure 6:**
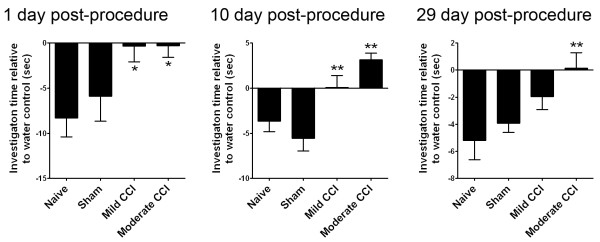
**Unilateral CCI results in deficits in olfactory avoidance behavior that recover at a rate proportional to CCI severity.** The duration of investigative behavior to a noxious acetic acid stimulus in relation to an odorless water control was recorded for each mouse within an open field apparatus during a 3-min testing period at 1, 10 and 29 days post-procedure. The data is plotted as the total time each mouse spent investigating the water-scented paper subtracted from the value obtained with the acetic-acid odorant to give the relative avoidance time (negative values indicate higher preference for the water control odor). The results reveal an acute effect of CCI on olfactory preference. At 1 day post-procedure, CCI mice (but not shams or naïve) show no preference for water over acetic acid (* p < 0.05 versus naïve). Lack of olfactory discrimination in this test persists in CCI mice at 10 days post-procedure (** p < 0.01 moderate CCI versus naïve, ** p < 0.01 mild CCI versus sham). At 29 days post-procedure, moderate CCI mice continue to show no preference in this test (** p < 0.01 moderate CCI versus naïve), whereas mild CCI mice show a preference for water over acetic acid similar to sham and naïve controls). Data shown as mean ± SEM with n = 10/group.

We next determined if deficits in olfactory bulb neurogenesis were associated with changes in olfactory-related behaviors by measuring the responses of mice to the noxious odor of acetic acid. This behavior test uses open-field video-tracking to record the behavior of mice toward a piece of filter paper soaked in either acetic acid or water (used as an odorless control). Mice were scored for the relative duration of investigative behavior of the acetic acid or water source (close proximity, sniffing behavior). Baseline measurements demonstrated a robust preference for investigating the water compared to the acetic acid-impregnated filter paper in support of the hypothesis that the acetic acid odor is adverse to the mice and stimulates an active avoidance. One day after the procedure, mild and moderate CCI mice displayed a clear difference in behavior in this test showing no preference to either the acetic acid or water. This was in stark contrast to naïve and sham controls which demonstrated a strong aversion to the acetic acid scent (p < 0.05, Figure 6). Lack of water over acetic acid preference remained when mild and moderate CCI mice were tested at 10 days post-procedure. Indeed CCI mice registered positive values at this stage of testing indicating a slight preference for investigating the acetic acid scent over water (Figure 6). As before, naïve and sham controls continued to avoid the acetic acid odor in this test, arguing against any habituation effect and confirming statistical differences with CCI mice (p < 0.01 moderate CCI, p < 0.01 mild CCI versus sham only, Figure 6). By 29 days post-procedure, mild CCI mice had reestablished behaviors consistent with avoidance of the acetic acid scent (no significant difference with either naïve or sham groups), whereas moderate CCI mice showed a continued lack of preference that was significantly different from naïve (p < 0.01, Figure 6). These data demonstrate focal cortical contusion impairs olfactory avoidance behavior in mice. The data reveal a post-injury recovery of this behavior that occurs at a rate relative to the severity of CCI with the less severe mild CCI recovering before moderate CCI mice.

Taken together, our analyses demonstrate unilateral, focal contusion to the somatosensory cortex impairs sensorimotor coordination and olfactory avoidance behavior in adult mice. These behavioral changes are accompanied by increased cell proliferation and decreased olfactory bulb neurogenesis in the ipsilateral cerebral hemisphere.

## Discussion and conclusions

In this study we demonstrated that a unilateral contusion to the left somatosensory cortex decreased neurogenesis in the olfactory bulb of the cerebral hemisphere ipsilateral to the injury. Decreases in olfactory bulb neurogenesis were recorded in mice subjected to a moderate 2 mm and mild 1 mm depth controlled cortical impact (CCI) at 30 days post-CCI. Chronic deficits in olfactory bulb neurogenesis induced by CCI were in contrast to the acute pro-proliferative effects of CCI in the SVZ neurogenic niche at 3 days post-CCI. Hence combined stereological analysis of adult SVZ and olfactory bulb cell populations reveal CCI is capable of stimulating proliferation in the SVZ neurogenic niche but this increase does not result in a corresponding increase in olfactory bulb neurogenesis.

One explanation for the failure of injury-induced neuroblasts to increase olfactory bulb neurogenesis is that cortical contusion disrupts the normal maturational process from the SVZ to the olfactory bulb. Neuroblasts born in the adult SVZ undergo chain migration within the RMS en route to the olfactory bulb where they switch to radial migration to reach destinations in the granular cell or periglomerular layers to form GABAergic interneurons [22,23]. Gross interruptions in the RMS by the CCI lesion cavity could account for the failure of migrating neuroblasts to reach olfactory bulb targets in moderate CCI mice in which the contusion resulted in a cavity extending through the RMS into the lateral ventricle in all mice analyzed at 30 days post-injury. However, the lesion was not as consistently extensive in mild CCI mice at 30 days post-injury – in almost half of the mice analyzed the lesion was confined to the cortex, sparing the corpus callosum and underlying RMS - and yet mild CCI mice also exhibited substantial deficits in neurogenesis at this stage (1.63-fold reduction compared to a 1.87-fold reduction with moderate CCI). This suggests additional factors such as cell death from the toxic effects of neuroinflammation [24] or the ectopic recruitment of SVZ neuroblasts [25-28] to damaged cortical tissue areas contributed to the reduction in olfactory bulb neurogenesis.

Similarly, behavioral testing demonstrated CCI resulted in a corresponding impairment of olfactory avoidance behavior in adult mice. This suggests an association between CCI deficits in olfactory bulb neurogenesis and impaired olfactory avoidance behaviors in adult mice. However, impaired olfactory discrimination persisted in moderate but not mild CCI mice at 30 days post-CCI and yet neurogenesis was significantly reduced by both CCI procedures. Therefore, it is unlikely that decreases in neurogenesis can solely account for this impairment. Damage to additional structures - the olfactory nerve or olfactory processing areas such as the amygdala, hippocampus and hypothalamus - may also contribute to this behavioral deficit.

In addition to injury effects on olfactory bulb neurogenesis and olfactory-related behaviors, the graded cortical contusion injury produced here resulted in unconditioned locomotor activity, deficits in motor coordination, increased macrophage/microglia cell proliferation in peri-contusional areas and chronic astrogliosis that were commensurate with lesion severity. These results are consistent with the electromagnetic CCI device producing a reproducible and graded contusion depth and graduated outcomes. Although there is good evidence that craniotomy alone in sham mice produces injury-associated effects [29], we were able to distinguish between the histological effects of two contusion depths differing by 1 mm with as few as 4 mice per group using unbiased stereological cell counting procedures. In behavior studies group sizes of 10 or 12 were sufficient to identify behavioral differences between the two CCI depths. This degree of injury reproducibility is comparable to published data using the same CCI device with accelerating rotarod testing in which performance differences were distinguishable between 0.5 mm changes in CCI depth with a group size of 12 (CCI at 2.7 mm left lateral and +3 mm Lambda ≈ -1.2 mm Bregma, see [9]).

Recently, two studies of graded CCI severity to the left parietal lobe have revealed increasing cognitive and emotional deficits with increasing CCI severity [30,31]. Spatial learning and memory deficits were most closely related to CCI severity in both studies, as would be expected from the location of their CCI directly superior to the hippocampus (CCI between Bregma and Lambda anatomical landmarks). In contrast, neither study observed the CCI-induced hyperactivity in open-field testing we detected. We speculate differences in locomotor behavior could result from differences in the rostral-caudal location of the CCI (left lateral at Bregma in our study versus between Bregma and Lambda). Thus, CCI studies consistently demonstrate the high comparative value of this approach in TBI research but also highlight the need for the detailed spatial definition of the contusion injury in comparative analysis.

## Competing interests

The authors disclose that no competing financial interests exist.

## Authors’ contributions

KLR carried out the animal surgeries, immunohistochemistry, behavioral testing, cell and lesion volume quantification, data analysis and editing of the manuscript. KJY and QZ assisted with tissue preparation and immunohistochemistry. MLD conceived of the study, participated in its design and coordination and drafted the manuscript. All authors have read and approved the final manuscript.
